# Phylogenetic ecology of gall crabs (Cryptochiridae) as associates of mushroom corals (Fungiidae)

**DOI:** 10.1002/ece3.1808

**Published:** 2015-11-24

**Authors:** Sancia E. T. van der Meij, Charles H. J. M. Fransen, Leon R. Pasman, Bert W. Hoeksema

**Affiliations:** ^1^ Naturalis Biodiversity CenterDarwinweg 22333 CRLeidenThe Netherlands; ^2^ Oxford University Museum of Natural HistoryParks RoadOxfordOX1 3PWUK

**Keywords:** Cospeciation, *Dacryomaia*, *Fungicola*, host specificity, Indo‐Pacific, new associations

## Abstract

Coral‐associated fauna is a relatively understudied topic. Hence, the nature of the relationship between an associated organism and its host is usually unknown. In the present study, the obligate associations between gall crabs (Decapoda: Cryptochiridae) and mushroom corals (Scleractinia: Fungiidae) are reviewed from a phylogenetic perspective. Based on field surveys, examination of museum material and a literature review, a total of 35 fungiid species have been found that act as hosts for four gall crab species. Fungiid‐associated gall crabs appear to be more geographically widespread than previously known, with new records showing their occurrences from the Red Sea and western Indian Ocean all the way to the central Pacific Ocean. The obligate nature of the association between cryptochirids and their hosts makes them an ideal model taxon to test for possible cospeciation events. The congruence between their phylogenies was tested by using the program Jane 4.0, resulting in cospeciation and duplication events between the crabs and their host corals. The sharing of several closely related host coral species by the same gall crab species or genus may provide support to models indicating phylogenetic relationships within the Scleractinia.

## Introduction

The integration of molecular analyses with skeleton microstructure data in recent phylogeny reconstructions of stony corals (Scleractinia) has initiated great opportunities in the advancement of scleractinian systematics (i.e., Benzoni et al. 2007; Budd et al. 2012; Arrigoni et al. 2014a,b; Huang et al. 2014). For the mushroom coral family Fungiidae, this approach has resulted in various changes at genus level and the inclusion of two additional species (Gittenberger et al. 2011; Benzoni et al. 2012). Fungiidae occur in the Indo‐Pacific with a distribution ranging from the Red Sea and eastern Africa to the west coast of central America (Hoeksema 1989). Several species have been recorded to live in association with fungiids. Most of the associated fauna consists of crustaceans and molluscs, but since recent discoveries it is also known to include acoel flatworms, ctenophores, polychaetes, hydroids, and various fishes (e.g., Hoeksema et al. 2012, 2013; Hoeksema and ten Hove 2014; Bos and Hoeksema 2015; van der Meij 2015a; Montano et al. 2015).

Gall crabs (Cryptochiridae) are obligate associates of stony corals, living in dwellings inside their coral hosts. They are common inhabitants of coral reefs, which are easily overlooked because of their small size and hidden life inside their coral hosts (Hoeksema and van der Meij 2013). Traditionally, gall crab genera used to be defined by host specificity (Fize and Serène 1957); a scheme that worked for some crab genera but proved to be unreliable for other genera (Kropp and Manning 1987). According to the last taxonomic revision of Indo‐Pacific gall crabs (Kropp 1990) only two species are known to live in association with mushroom corals: *Fungicola fagei* (Fize and Serène 1956a) and *Fungicola utinomi* (Fize and Serène 1956a). Hoeksema et al. (2012) reported on a *Dacryomaia* species as a third cryptochirid species associated with Fungiidae, whereas van der Meij and Hoeksema (2013) reported on a fourth species. The latter concerned a cryptic species closely related to *F*.* fagei*, recently described as *Fungicola syzygia* van der Meij 2015a.

The obligate nature of the association between cryptochirids and their hosts raises questions about possible cospeciation between the two. Studies on the associated fauna of stony corals, however, have so far largely been focused on the symbiont without information on the host coral. In this study, the following questions are addressed. Is there an overlap between the geographical distribution of the corals and their associated gall crabs? Are common coral species more likely to be inhabited by gall crabs than less commonly occurring coral species? Are the phylogenetic relationships of the host corals reflected in the phylogenetic relationships of the crabs; hence, is there some kind of cospeciation between the two?

To answer these questions, fungiid‐associated gall crabs were studied from the perspective of the host by collecting crabs from as many coral species as possible. Fieldwork in various parts of the Indo‐Pacific, examination of museum collections, and a review of available literature were carried out in order to obtain host, distribution, and occurrence records. The gall crab–coral associations and occurrence rates were projected on a cladogram of the Fungiidae in order to reconstruct the evolutionary history of the associations of the crabs and their host species. The congruence between the fungiid and gall crab phylogenies was tested for cospeciation events with the help of the program Jane 4.0.

## Material and Methods

### Historical records

In order to examine the distribution of fungiid‐associated gall crabs, the coral collections of Naturalis Biodiversity Center (RMNH) in Leiden, the Netherlands, and the Royal Belgian Institute of Natural Sciences (IRSNB) in Brussels, Belgium, were searched for the presence of gall crabs or their vacated pits. Additional records were obtained from the coral collections of the University of Milano‐Bicocca (UNIMIB) in Milan, Italy, and the AMNH (American Museum of Natural History) in New York, USA. Some pits contained (dried) gall crab carapaces, which were examined for identification (Table 1). Gall crab identifications were based on Fize and Serène (1957), Kropp (1990) and van der Meij (2015a), whereas coral identifications were based on Hoeksema (1989), Gittenberger et al. (2011) and Benzoni et al. (2012). Literature was studied to obtain further distribution records. Host species data provided by Fize and Serène (1957) were taken from the main text (p. 122, 130, 134, 156, 171) because these were assumed to be more correct than those listed on p. 13. In addition, a few field observations were included (photo vouchered).

**Table 1:** Distribution of gall crab species based on museum records of Fungiidae containing coral gall crabs (indicated by species name) or their pits (+), literature, and incidental observations (photo vouchers). Coral names updated according to Gittenberger et al. (2011). *Fungicola utinomi* without host record was reported from Indonesia (Moluccas – Kropp 1994) and Micronesia (Mariana Isl. – Paulay et al. 2003)

Coral host	Museum records	Localities	Reference for locality data
*Cycloseris costulata* (Ortmann, 1889)	*Fungicola syzygia* ^1^	B, G, J, S	
*Cycloseris curvata* (Hoeksema, 1989)	+^3^	C	
*Cycloseris cyclolites* (Lamarck, 1815)	+^1^	F, G	
*Cycloseris fragilis* (Alcock, 1893)	+^1^	G	
*Cycloseris mokai* (Hoeksema, 1989)	+^1^	G	
*Cycloseris sinensis* M. Edwards and Haime, 1851	+^1^	G	
*Cycloseris tenuis* (Dana, 1846)	+^1^	F, K	
*Danafungia horrida* (Dana, 1846)	−	H	Fize and Serène 1957 (*Fungicola utinomi*)
*Fungia fungites* (Linnaeus, 1758)	+^1^, *F. utinomi* ^2^	G, H, M	Fize and Serène 1957 (*F. utinomi*)
*Herpolitha limax* (Esper, 1797)	−	O	This study
*Lithophyllon concinna* (Verrill, 1864)	+^1^	G, K	
*Lithophyllon ranjithi* Ditlev, 2003	+^1^	J	
*Lithophyllon repanda* (Dana, 1846)	+^1^, *F. utinomi* ^2^	H, K, M, N, O	Takeda and Tamura 1979 (*F. utinomi*); Fize and Serène 1957 (*F. utinomi*); this study
*Lithophyllon scabra* (Döderlein, 1901)	*Dacryomaia* sp., *Fungicola* sp.	G	
*Lithophyllon undulatum* Rehberg, 1892	*Dacryomaia* sp.	G, I	This study
*Lobactis scutaria* (Lamarck, 1801)	*Fungicola* sp.^4^	R	
*Pleuractis granulosa* (Klunzinger, 1879)	*F*.* syzygia* ^1^ ^,^ ^2^	A, E, G, L, P, S	
*Pleuractis gravis* (Nemenzo, 1956)	*F*.* syzygia* ^1^	G	
*Pleuractis moluccensis* (Van der Horst, 1919)	+^1^	G, K	
*Pleuractis paumotensis* (Stutchbury, 1833)	*F*.* syzygia* ^1^ ^,^ ^1^	E, G, H, N, O, Q, S	Fize and Serène 1957 (?*F*.* syzygia*); Takeda and Tamura 1979 (?*F*.* syzygia*); this study
*Pleuractis seychellensis* (Hoeksema, 1993)	*F*.* syzygia* ^1^	D	
*Pleuractis taiwanensis* (Hoeksema and Dai, 1991)	+^1^	G	
*Podabacia crustacea* (Pallas, 1766)	*Fungicola fagei* ^1^	G	
*Podabacia motuporensis* Veron, 1990	+^1^	L	
*Podabacia sinai* Veron, 2000	*F*.* fagei* ^1^	L	
*Sandalolitha dentata* Quelch, 1884	+^1^	G, H	Fize and Serène 1957 (*F*.* fagei*)
*Sandalolitha robusta* (Quelch, 1886)	*F*.* fagei* ^1^ ^,^ ^2^	M, S	

Localities of the listed host species: A = Israel (Eilat, Red Sea); B = Kenya (western Indian Ocean); C = Gulf of Aden, Yemen; D = Seychelles (western Indian Ocean); E = Maldives (central Indian Ocean); F = Thailand (Phuket); G = Indonesia; H = Vietnam (Nha Trang); I = Malaysia (Tioman Isl.); J = Malaysia (Sabah); K = Taiwan; L = Palau; M = Papua New Guinea (Bismarck Sea); N = Japan (Yaeyama Isl.); O = Australia (GBR, off Cairns); P = Samoa Isl. (western Pacific Ocean); Q = Tahiti (central Pacific Ocean); R = Hawaii; S = Vanuatu.

Museum records: ^1^RMNH, ^2^IRSNB, ^3^UNIMIB, ^4^AMNH. In bold, localities based on literature references and/or incidental observations.

### Fieldwork

A large part of the fieldwork was carried out in Spermonde Archipelago – SW Sulawesi (Indonesia), in the southern part of the Makassar Strait (1994), where belt quadrats of 50 × 2 m^2^ were used to study gall crab – fungiid occurrences at 11 sites (see Hoeksema 2012a,b). Per quadrat the density of mushroom coral species and the percentage of inhabited corals were recorded. Transect work was mostly carried out on the western reef slopes as mushroom coral species are most abundant at these sides of the reefs, which are the most exposed to wind and wave action. In addition, inhabited mushroom corals were collected to obtain the gall crab specimens. The corals were split by use of a hammer and chisel and coral fragments containing the gall crabs were immersed in 80% ethanol for at least 1 h to immobilize the crabs, which were subsequently transferred to labeled vials.

Further data on fungiid–gall crab associations were collected during fieldwork (2007–2012) in Indonesia (Raja Ampat – W Papua, Bunaken – N Sulawesi, Ternate – N Moluccas, Lembeh Strait – N Sulawesi) and Malaysia (Semporna – N Borneo, Kudat – N Borneo). Mushroom corals from various reef sites were sampled for gall crabs, attempting to sample as many host species as possible from deep to shallow reef zones. Mushroom corals containing gall crabs were collected until a representative collection of the Fungiidae species was reached. The corals were sampled in the same way as described above after being photographed with a Canon 400D camera (Canon Inc, Tokyo, Japan) equipped with a 50 mm Sigma macro‐lens (Sigma Corporation, Bandai, Japan). All gall crab specimens are deposited in the collections of Naturalis in Leiden, the Netherlands (collection coded as RMNH.Crus.D).

Additional records were obtained from Vietnam (Nha Trang – 2006), Australia (Great Barrier Reef – off Cairns (2010), New Caledonia (2012, Loyalty Is. – 2013), Malaysia (Payar Isl, Tioman Isl – 2013), and the Maldives (Faafu Atoll – 2014).

### Cophylogenetic analyses based on host association data

The phylogenetic congruence of hosts and associates was tested by using the program Jane 4.0 (Conow et al. 2010), based on the coral phylogenies in the studies of Gittenberger et al. (2011) and Benzoni et al. (2012), and the gall crab phylogeny presented by van der Meij (2015a). The phylogeny reconstruction of the Fungiidae by Gittenberger et al. (2011) was based on combined and separate ITS and COI datasets. This reconstruction was carried out in PAUP 4.0 (Swofford 2002) for MP (maximum parsimony) and neighbor joining analyses and in MrBayes 3.0 (Ronquist and Huelsenbeck 2003) for a BI (Bayesian inference) analysis. In the study by Benzoni et al. (2012), the position of *Cycloseris explanulata* and *Cycloseris wellsi* was inferred based on COI and a selection of nuclear rDNA (ITS1, 5.8S, ITS2, 18S and 28S). Phylogenetic relationships were reconstructed using BI, MP, and ML (maximum likelihood). The phylogeny of the fungiid‐associated cryptochirids by van der Meij (2015a) was reconstructed based on COI, 16S, and H3 sequence data, using a BI analysis.

The program Jane is based on an event‐based model which considers cospeciation as the most parsimonious explanation for congruence between host taxa and associate taxa trees. Detection of coevolutionary relationships is easily obstructed by the complex interplay of events, that is, cospeciation, duplication (intrahost speciation), host switching, sorting (extinction), and inertia (lack of parasite speciation). For definitions, we refer to Paterson and Banks (2001) and Conow et al. (2010). The evolutionary events are used to superimpose the phylogeny reconstruction of the associated taxon on that of the host taxon. Jane 4.0 assigns a cost to each evolutionary event, after which it seeks to find mappings minimizing the total cost. The default costs settings of Jane were used, as follows: cospeciation (0), duplication (1), duplication – host switching (2), loss (1), and failure to diverge (1). Statistical analyses are performed by comparing the optimal (minimum) costs found for the host parasite dataset against randomized datasets (Cruaud et al. 2012).

The program can take multiple host associations into account, but occurrence levels are not supported, and therefore, the program was run twice: (1) on the complete dataset including all host specificity data; (2) on a dataset comprising only the common hosts (see Norton and Carpenter 1998). In this second dataset, sporadic host occurrences (singletons) were removed. In both runs, the following settings were used (stats mode): 100 generations, population size 500, and sample size 100. All other settings were left unchanged.

## Results

### Distribution based on historical records

Based on museum and literature records, the distribution of Fungiidae‐associated gall crabs ranges from Eilat in the Red Sea, and Kenya in the western Indian Ocean, toward Hawaii and Tahiti in the central Pacific Ocean (Table 1).

### Occurrence records

**Figure 1:**
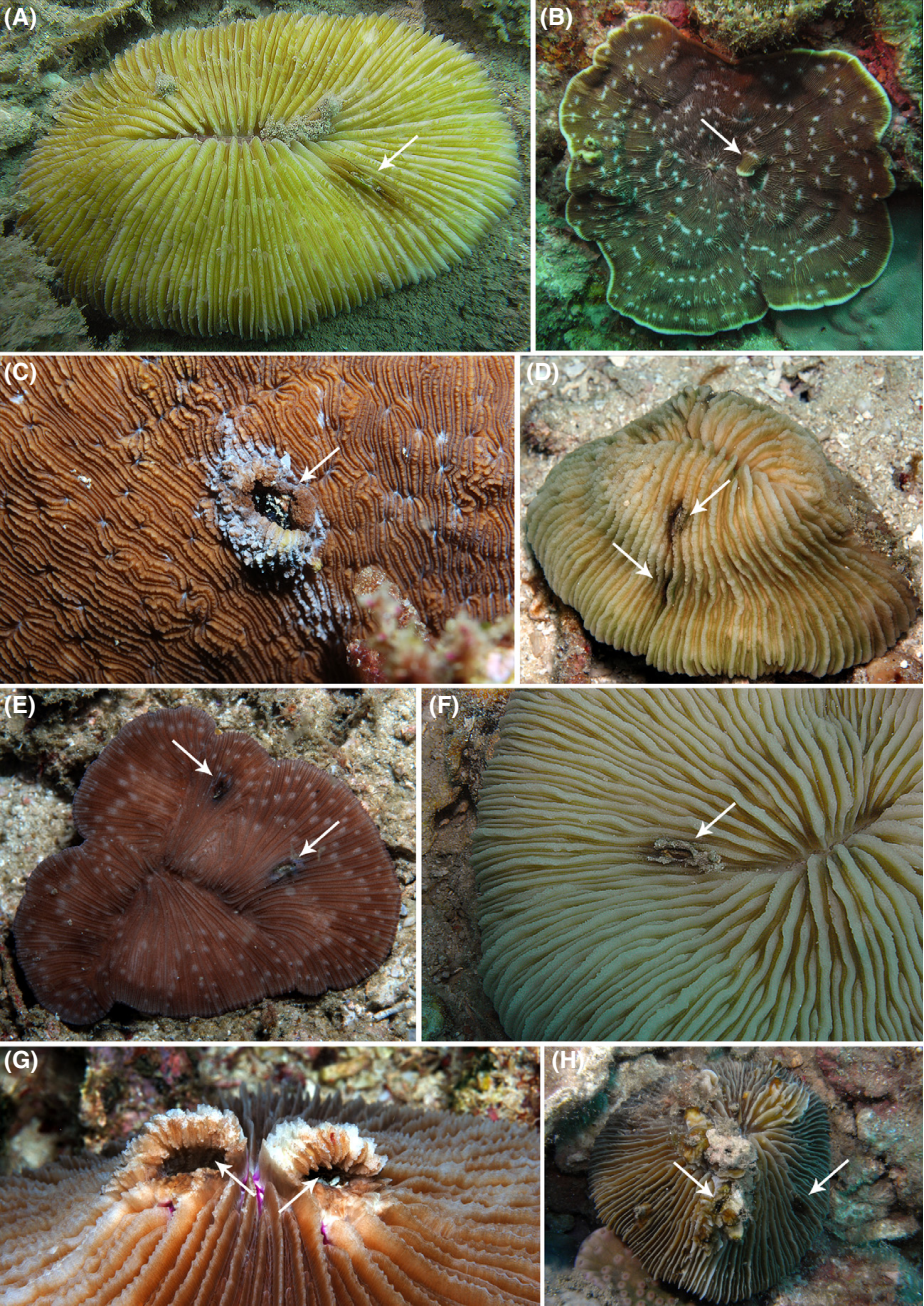
Mushroom corals with gall crab dwellings (arrows). (A) *Pleuractis paumotensis* (Nha Trang, Vietnam); (B) *Lithophyllon undulatum* (Nha Trang, Vietnam); (C) *Podabacia crustacea* (Raja Ampat, Indonesia); (D) *Pleuractis moluccensis* (Nha Trang, Vietnam); (E) *Cycloseris sinensis* (Raja Ampat, Indonesia); (F) *Pleuractis granulosa* (Ternate, Indonesia); (G) *Lithophyllon repanda* (Raja Ampat, Indonesia); (H) *Lithophyllon scabra* (Nha Trang, Vietnam). Photographs not to scale.

**Figure 2:**
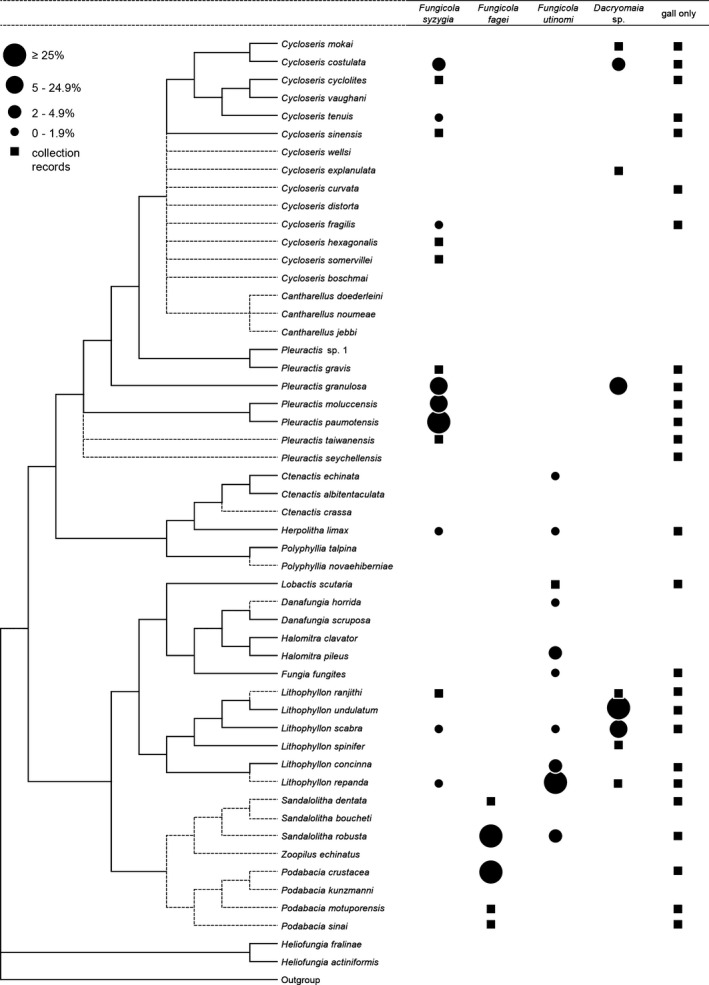
Cladogram of the Fungiidae (based on Gittenberger et al. 2011; Benzoni et al. 2012), combined with gall crab associations. Percentages (depicted as filled circles) portray how the gall crabs are distributed over their coral hosts: *Fungicola syzygia* (*n* = 316), *Fungicola fagei* (*n* = 4), *Fungicola utinomi* (*n* = 82), and *Dacryomaia* sp. (*n* = 29), based on fieldwork in SW Sulawesi using belt quadrats. Other records (depicted as filled squares) are based on collection data (Table 1) and fieldwork other than SW Sulawesi (see Material and Methods).

Data on crab occurrences obtained from the belt quadrats in the Spermonde Archipelago are projected on a cladogram of the Fungiidae (Table 2, Fig. 2). Percentages per host species are based on the number of encountered coral specimens per gall crab species. Figure 1 shows gall crab dwellings in eight of their common gall crab hosts. *Fungicola fagei* was only found inhabiting corals belonging to the genera *Podabacia* and *Sandalolitha*, and *F*.* syzygia* was predominantly found in corals of the genus *Pleuractis* and to a lesser extent in *Cycloseris*, whereas *F. utinomi* was predominantly found in *Lithophyllon repanda*. *Dacryomaia* sp. mainly inhabits corals of the genera *Lithophyllon*, and was primarily associated with *Lithophyllon undulatum*. To a lesser extent, it also occurs in the genera *Cycloseris* and *Pleuractis*. In the belt quadrats, only one specimen of *Dacryomaia* sp. was recorded from the genus *Cycloseris*.

**Table 2:** Mushroom coral species (Fungiidae) acting as host for gall crab species in the Spermonde Archipelago, SW Sulawesi. The number of collected coral specimens hosting specified gall crab species is given

Coral host	*Fungicola syzygia*	*Fungicola fagei*	*Fungicola utinomi*	*Dacryomaia* sp.
*Ctenactis echinata* (Pallas, 1766)			1	
*Cycloseris costulata* (Ortmann, 1889)	8			1
*Cycloseris fragilis* (Alcock, 1893)	2			
*Cycloseris tenuis* (Dana, 1846)	1			
*Danafungia horrida* (Dana, 1846)			1	
*Fungia fungites* (Linnaeus, 1758)			1	
*Halomitra pileus* (Linnaeus, 1758)			3	
*Herpolitha limax* (Esper, 1797)	1		1	
*Lithophyllon concinna* (Verrill, 1864)			4	
*Lithophyllon repanda* (Dana, 1846)	1		68	
*Lithophyllon scabra* (Döderlein, 1901)	1		1	7
*Lithophyllon undulatum* Rehberg, 1892				15
*Pleuractis granulosa* (Klunzinger, 1879)	49			6
*Pleuractis moluccensis* (Van der Horst, 1919)	40			
*Pleuractis paumotensis* (Stutchbury, 1833)	213			
*Podabacia crustacea* (Pallas, 1766)		1		
*Sandalolitha robusta* (Quelch, 1886)		3	2	

### Host associations and cophylogenetic analyses

The total number of Fungiidae that are host to gall crabs is 35 (Fig. 2, Table S1). *Fungicola utinomi* is found to be associated with 10 mushroom coral species, *F*.* fagei* with five fungiids, and *F*.* syzygia* with 15 host corals. *Dacryomaia* sp. appears to be associated with nine fungiid species (Fig. 2, Table 2). *Cycloseris curvata* and *C*.* explanulata* are new host records. Hoeksema et al. (2012) also recorded *Polyphyllia talpina* as a gall crab host. Further inspection of the material in the Naturalis collections revealed that the earlier reported dwelling is likely not from a gall crab, because the two pits in the host coral are interconnected and the surface of the dwelling is not smooth. These characteristics argue against a gall crab dwelling, and we therefore remove this coral species from the list of fungiid gall crab hosts until more evidence becomes available.

Based on the analysis in Jane 4.0 on the complete dataset (Fig. S2), there are two duplication events, one cospeciation event, 34 losses, and 37 failures to diverge. The smaller dataset (Fig. S4), comprised of only the common hosts, resulted in one duplication event, one duplication plus host switch event, one cospeciation event, 20 losses, and 11 failures to diverge. Both results show that the costs of the random sample solutions are higher than the optimal [=cospeciation] solution (Figs S3, S5).

## Discussion

Invertebrate taxa account for the greatest numerical abundance and diversity of species on coral reefs, yet have received rather little attention (Netchy et al. in press). Our awareness of coral reef ecosystem functioning is derived from what we know about a relative small proportion of coral reef species. Animals so closely associated with their habitat may be vital to the maintenance of critical ecological systems pertaining to coral health (Stella et al. 2010), and as such could be potentially useful as environmental indicators (Thomas 1993; Scaps and Denis 2008). In this study, we used a phylogeny reconstruction of the Fungiidae corals to map host associations and occurrence rates. Using phylogeny models to map ecologically meaningful traits of species is a fusion between ecology and evolution, also known as phylogenetic ecology or phylo‐ecology (Westoby 2006; Hoeksema 2012a).

### Distribution records

Until the late 1960s, the genus *Fungicola* was only known from Vietnam and since then, just a few records became available from elsewhere (Takeda and Tamura 1979; Kropp 1990, 1994). van der Meij and Hoeksema (2013) and van der Meij (2015a) added several new records of the genus in Indonesia and Malaysia. The present research on museum collections resulted in the availability of many additional records for all three *Fungicola* species (Table 1). During a short survey on the Great Barrier Reef off Cairns in May 2010, one specimen of *F. utinomi* was observed in *L. repanda*, and individuals of *Fungicola* sp. were observed in *Pleuractis paumotensis* and *Herpolitha limax*. *Fungicola syzygia* is now reported from the Red Sea and Kenya in the west, to Japan and Vanuatu in the east, while *F*.* fagei* and *F. utinomi* are now recorded from Vietnam and Indonesia in the west, to Japan and possibly Australia (GBR) in the east. *Dacryomaia* sp. is recorded from the heart of the Coral Triangle: Indonesia and Malaysia (Tables 1, 2). The Indo‐Pacific mushroom coral *Lobactis scutaria*, host to *F. utinomi*, was introduced to Jamaica from Eilat in 1966 (by man) and has since established an apparently viable population (Bush et al. 2004). So far, no gall crabs have been reported for this population.

Hoeksema and Gittenberger (2008) reported that coral gall crabs appear to be abundant in Nha Trang, Vietnam, especially in *Podabacia crustacea* and *L. repanda*. Based on their results, the gall crab fauna in Vietnam likely consists of *F*.* fagei* and *F. utinomi*, which is in agreement with the reports by Fize and Serène (1956a, 1957). According to Takeda and Tamura (1979) *F. utinomi* is more common in Japan than *F*.* fagei* of which only two records/specimens are known. Based on van der Meij (2015a), the identification of *F*.* fagei* by Takeda and Tamura (1979) should most likely be corrected to *F*.* syzygia*. The main hosts of *F*.* fagei* are, however, also present in Japan (Hoeksema 1989). It is unclear whether the findings of Takeda and Tamura (1979) are caused by undersampling of particular species of mushroom coral hosts or by lower occurrence rates of *F*.* fagei* and *F*.* syzygia*. The genus *Dacryomaia* has been recorded from nonfungiid corals at the Ryukyu Islands (Japan), Caroline Isl. (Kiribati), Guam, and other Mariana Isl. (Table 1); however, these records most likely concern *D*.* japonica*,* D*.* edmonsoni*, and/or further undescribed species (Paulay et al. 2003; S. E. T. van der Meij unpubl. data).

There appears to be much overlap in the geographical distribution of the mushroom corals and fungiid‐associated gall crabs (Hoeksema 1989; Table 2). The distribution ranges of the gall crab species is likely to be more extensive than reported in this study. Presumably rare species, or species with a disjunct distribution, may be represented in scientific coral collections without being noticed. This confirms the value of historical collection material for biogeographical research, since museum specimens may show that species display a greater distribution range than previously assumed (Drew 2011; Hoeksema et al. 2011; van der Meij and Visser 2011; Hoeksema in press).

### Occurrence records

The results of the belt quadrats study in the Spermonde Archipelago show that the percentage of encountered gall crabs appears to be linked to the relative occurrence of their host corals. The coral species for which most gall crabs are reported are also among the most widespread and commonly occurring mushroom corals, that is, *L. repanda*,* Pleuractis granulosa*,* Pleuractis moluccensis,* and *P*.* paumotensis* (see Hoeksema 2012b). However, some common mushroom corals are not frequently inhabited by gall crabs (e.g., *Halomitra pileus*,* L. scutaria*,* Sandalolitha dentata*), whereas others appear to be associated with one or more species (Table 2). Small and/or thin species (e.g., *Cycloseris boschmai*,* C*.* distorta*,* Halomitra clavator*,* Zoopilus echinatus*), those with fleshy polyps and permanently extending tentacles (e.g., *Heliofungia* spp., *Polyphyllia* spp.), or rarely observed species (e.g., *Cantharellus* spp., *Podabacia kunzmanni*,* Sandalolitha boucheti*) are not yet found to be associated with gall crabs.

### Host associations and cophylogenetic analyses

The total number of fungiid species inhabited by gall crabs is now 35 (Table S1). *Cycloseris explanulata* and *C*.* wellsi*, both encrusting, thin species, were previously not yet included in the Fungiidae (prior to Benzoni et al. 2012). During most of the present research, they were therefore not considered as potential host for fungiid‐associated gall crabs. This likely lead to undersampling of these coral hosts. *Polyphyllia talpina* is no longer considered to be a gall crab host. This is in line with previous observations that gall crabs are mostly not observed in coral species with fleshy polyps and large tentacles (e.g., van der Meij 2014; but see van der Meij 2015a).

Recently, the coral family Fungiidae was revised based on a molecular analysis (Gittenberger et al. 2011). The majority (95%) of *F*.* syzygia* specimens was encountered in corals of some *Pleuractis* species, that is, *P*.* paumotensis*,* P*.* granulosa*, and *P*.* moluccensis* (Fig. 2). Apart from the genus *Pleuractis*, this gall crab species also occurs in the closely related genus *Cycloseris*. *Fungicola utinomi* is in almost all cases associated with *L. repanda*, but occurs to a lesser extent in corals belonging to other genera. None of the inhabited fungiids were simultaneously occupied by more than one gall crab species, but one host species, *Lithophyllon scabra*, was found inhabited by three different gall crab species. The sporadic selection of certain corals as a host might be related to a low availability of the common or “preferred” host species at a certain locality. It might also be the result of a collecting artifact, as it remains possible that host associations have geographical variability.

*Dacryomaia* sp. mostly targets *L. undulatum*, and to a lesser extent *L*.* scabra*, and *P. granulosa*. Other species in the genus *Dacryomaia* are associated with the genera *Coscinaraea* (Coscinaraeidae), *Leptastrea* (Scleractinia *incertae sedis*), and *Psammocora* (Psammocoridae) (Kropp 1990; van der Meij, unpubl. data). This is likely not a coincidence, since these genera are closely related with Fungiidae (Fukami et al. 2008; Kitahara et al. 2010; Huang 2012). The genus *Dacryomaia*, which contains undescribed species, is in need of a taxonomic revision (Paulay et al. 2003; van der Meij, unpubl. data). Further research on the gall crabs of this genus and their host associations may be used to verify congruencies of the phylogenetic relationships of the associated fauna and their hosts as support for reconstructed phylogenetic relationships within the Scleractinia.

The analyses in Jane 4.0 show that there have been cospeciation and duplication events between fungiids and their gall crab inhabitants, as well as several losses and failures to diverge. Differences between the outcomes of the analysis on the complete dataset versus the common host dataset can be explained by the settings of the program Jane. Associations between host and symbiont are not weighed; hence, single recorded associations are given the same value in the analysis, obscuring the overall patterns between host and symbiont. Both analyses show that even within a moderately small coral family like the Fungiidae with just over 50 species (Gittenberger et al. 2011; Benzoni et al. 2012), four gall crab associates occupy their own niche and are host‐specific to a certain degree. *Fungicola fagei* appears to be stricter in its host associations than the other three fungiid‐associated species.

Gall crabs are mostly host specific on coral genus level, which explains the high number of losses and failures to divergence in the current Jane analysis based on Fungiidae‐associated cryptochirids alone. A large‐scale phylogeny reconstruction of the family Cryptochiridae and their scleractinian hosts provides more insight into the cospeciation between these associates and their hosts (van der Meij 2015c). The relationship between Scleractinia and Cryptochiridae appears to be so tight that gall crabs can be used as phylogenetic indicators of scleractinian evolution (van der Meij 2015c), which contradicts the hypothesis of Kropp and Manning (1987) that the generic identity of coral hosts is an unreliable character for defining gall crab genera. Further research is needed to determine whether the observed cospeciation events between the crabs and the host corals occurred at the same point in time or whether the gall crabs diversified at a later stage than their host corals.

## Conflict of Interest

None declared.

## Supporting information


**Table S1**. References to literature mentioning fungiid hosts of gall crabs.

**Figure S1.** Tree resulting from analysis in Jane 4.0 showing the different coevolutionary events between Fungiidae (black lines) and Cryptochiridae (blue lines), based on the complete dataset.

**Figure S2.** Histogram resulting from a stats run in Jane 4.0 on the complete dataset, showing the distributions of costs of the random sample solutions. The costs of the optimal [=coevolution] solution is indicated by the red dotted line.

**Figure S3.** Tree resulting from analysis in Jane 4.0 showing the different coevolutionary events between Fungiidae (black lines) and Cryptochiridae (blue lines), based on the common occurrences dataset.

**Figure S4.** Histogram resulting from a stats run in Jane 4.0 on the common occurrences dataset, showing the distributions of costs of the random sample solutions. The costs of the optimal [=coevolution] solution is indicated by the red dotted line.

## References

[ece31808-bib-0001] Arrigoni, R. , Y. F. Kitano , J. Stolarski , B. W. Hoeksema , H. Fukami , F. Stefani , et al. 2014a A phylogeny reconstruction of the Dendrophylliidae (Cnidaria, Scleractinia) based on molecular and micromorphological criteria, and its ecological implications. Zool. Scr. 43:661–688.

[ece31808-bib-0002] Arrigoni, R. , Z. T. Richards , C. A. Chen , A. H. Baird , and F. Benzoni . 2014b Taxonomy and phylogenetic relationships of the coral genera *Australomussa* and *Parascolymia* (Scleractinia, Lobophylliidae). Contrib. Zool. 83:195–215.

[ece31808-bib-0003] Benzoni, F. , F. Stefani , J. Stolarski , M. Pichon , G. Mitta , and P. Galli . 2007 Debating phylogenetic relationships of the scleractinian *Psammocora*: molecular and morphological evidences. Contrib. Zool. 76:35–54.

[ece31808-bib-0004] Benzoni, F. , R. Arrigoni , F. Stefani , B. T. Reijnen , S. Montano , and B. W. Hoeksema . 2012 Phylogenetic position and taxonomy of *Cycloseris explanulata* and *C*.* wellsi* (Scleractinia: Fungiidae): lost mushroom corals find their way home. Contrib. Zool. 81:125–146.

[ece31808-bib-0005] Bos, A. , and B. W. Hoeksema . 2015 Cryptobenthic fishes and coinhabiting shrimps associated with the mushroom coral *Heliofungia actiniformis* (Fungiidae) in the Davao Gulf, Philippines. Environ. Biol. Fishes 98:1479–1489.

[ece31808-bib-0006] Budd, A. F. , H. Fukami , N. D. Smith , and N. Knowlton . 2012 Taxonomic classification of the reef coral family Mussidae (Cnidaria: Anthozoa: Scleractinia). Zool. J. Linn. Soc. 166:65–529.

[ece31808-bib-0007] Bush, S. L. , W. F. Precht , J. D. Woodley , and J. F. Bruno . 2004 Indo‐Pacific mushroom corals found on Jamaican reefs. Coral Reefs 23:234.

[ece31808-bib-0008] Conow, C. , D. Fielder , Y. Ovadia , and R. Libeskind‐Hadas . 2010 Jane: a new tool for the cophylogeny reconstruction problem. Algorithms Mol. Biol. 5:16. 20181081 10.1186/1748-7188-5-16 PMC2830923

[ece31808-bib-0009] Cruaud, A. , N. Rønsted , B. Chantarasuwan , L. Siang Chou , W. L. Clement , A. Couloux , et al. 2012 An extreme case of plant–insect codiversification: figs and fig‐pollinating wasps. Syst. Biol. 61:1029–1047. 22848088 10.1093/sysbio/sys068 PMC3478567

[ece31808-bib-0010] Drew, J. 2011 The role of natural history institutions and bioinformatics in conservation biology. Conserv. Biol. 25:1250–1252. 22070276 10.1111/j.1523-1739.2011.01725.x

[ece31808-bib-0011] Fize, A. , and R. Serène . 1956a Note préliminaire sur huit espèces nouvelles, dont une d'un genre nouveau, d'Hapalocarcinidés. Bull. Soc. Zool. Fr. 45:375–378.

[ece31808-bib-0013] Fize, A. , and R. Serène . 1957 Les Hapalocarcinides du Viet‐Nam. Mém. Inst. Océanogr. Nhatrang 10:1–202, pls. 1–18.

[ece31808-bib-0014] Fukami, H. , C. A. Chen , A. F. Budd , A. Collins , C. Wallace , Y. Y. Chuang , et al. 2008 Mitochondrial and nuclear genes suggest that stony corals are monophyletic but most families of stony corals are not (Order Scleractinia, Class Anthozoa, Phylum Cnidaria). PLoS ONE 3:e3222. 18795098 10.1371/journal.pone.0003222 PMC2528942

[ece31808-bib-0015] Gittenberger, A. , B. T. Reijnen , and B. W. Hoeksema . 2011 A molecularly based phylogeny reconstruction of mushroom corals (Scleractinia: Fungiidae) with taxonomic consequences and evolutionary implications for life history traits. Contrib. Zool. 80:107–132.

[ece31808-bib-0016] Hoeksema, B. W. 1989 Taxonomy, phylogeny and biogeography of mushroom corals (Scleractinia: Fungiidae). Zool. Verh. 254:1–295.

[ece31808-bib-0017] Hoeksema, B. W. 2012a Evolutionary trends in onshore‐offshore distribution patterns of mushroom coral species (Scleractinia: Fungiidae). Contrib. Zool. 81:199–221.

[ece31808-bib-0018] Hoeksema, B. W. 2012b Distribution patterns of mushroom corals (Scleractinia: Fungiidae) across the Spermonde Shelf, South Sulawesi. Raffles Bull. Zool. 60:183–212.

[ece31808-bib-0019] Hoeksema, B. W. in press. Latitudinal species diversity gradient of mushroom corals off eastern Australia: a baseline from the 1970s. Estuar. Coast. Shelf Sci. doi: 10.1016/j.ecss.2015.05.015.

[ece31808-bib-0020] Hoeksema, B. W. , and A. Gittenberger . 2008 Records of some marine parasitic mollusks from Nha Trang, Vietnam. Basteria 72:129–133.

[ece31808-bib-0021] Hoeksema, B. W. , and H. A. ten Hove . 2014 First record of a Christmas tree worm in a mushroom coral (Loyalty Islands, Southwest Pacific). Coral Reefs 33:717.

[ece31808-bib-0022] Hoeksema, B. W. , and S. E. T. van der Meij . 2013 Gall crab city: an aggregation of endosymbiotic crabs inhabiting a colossal colony of *Pavona clavus* . Coral Reefs 32:59.

[ece31808-bib-0023] Hoeksema, B. W. , J. van der Land , S. E. T. van der Meij , L. P. van Ofwegen , B. T. Reijnen , R. W. M. van Soest , et al. 2011 Unforeseen importance of historical collections as baselines to determine biotic change of coral reefs. Mar. Ecol. 32:135–141.

[ece31808-bib-0024] Hoeksema, B. W. , S. E. T. van der Meij , and C. H. J. M. Fransen . 2012 The mushroom coral as a habitat. J. Mar. Biol. Assoc. U. K. 92:647–663.

[ece31808-bib-0025] Hoeksema, B. W. , Z. Waheed , and A. Alamaru . 2013 Out of sight: aggregations of epizoic comb jellies underneath mushroom corals. Coral Reefs 32:1065.

[ece31808-bib-0026] Huang, D. 2012 Threatened reef corals of the world. PLoS ONE 7:e34459. 22479633 10.1371/journal.pone.0034459 PMC3316686

[ece31808-bib-0027] Huang, D. , F. Benzoni , H. Fukami , N. Knowlton , N. D. Smith , and A. F. Budd . 2014 Taxonomic classification of the reef coral families Merulinidae, Montastraeidae, and Diploastraeidae (Cnidaria: Anthozoa: Scleractinia). Zool. J. Linn. Soc. 171:277–355.

[ece31808-bib-0028] Kitahara, M. , S. D. Cairns , J. Stolarski , D. Blair , and D. J. Miller . 2010 A comprehensive phylogenetic analysis of the Scleractinia (Cnidaria, Anthozoa) based on mitochondrial CO1 sequence data. PLoS ONE 5:e11490. 20628613 10.1371/journal.pone.0011490 PMC2900217

[ece31808-bib-0029] Kropp, R. K. 1990 Revision of the genera of gall crabs (Crustacea: Cryptochiridae) occurring in the Pacific Ocean. Pac. Sci. 44:417–448.

[ece31808-bib-0030] Kropp, R. K. 1994 The gall crabs (Crustacea: Decapoda: Brachyura: Cryptochiridae) of the Rumphius expeditions revisited, with descriptions of three new species. Raffles Bull. Zool. 42:521–538.

[ece31808-bib-0031] Kropp, R. K. , and R. B. Manning . 1987 The Atlantic gall crabs, family Cryptochiridae (Crustacea: Decapoda: Brachyura). Smithson. Contrib. Zool. 462:1–21.

[ece31808-bib-0032] van der Meij, S. E. T. 2014 Host species, range extensions, and an observation of the mating system of Atlantic shallow‐water gall crabs (Decapoda: Cryptochiridae). Bull. Mar. Sci. 90:1001–1010.

[ece31808-bib-0033] van der Meij, S. E. T. 2015a Host relations and DNA reveal a cryptic gall crab species (Crustacea: Decapoda: Cryptochiridae) associated with mushroom corals (Scleractinia: Fungiidae). Contrib. Zool. 84:39–57.

[ece31808-bib-0034] van der Meij, S. E. T. 2015b A new gall crab species (Brachyura, Cryptochiridae) associated with the free‐living coral *Trachyphyllia geoffroyi* (Scleractinia, Merulinidae). ZooKeys 500:61–72. 25987871 10.3897/zookeys.500.9244 PMC4432240

[ece31808-bib-0035] van der Meij, S. E. T. 2015c Adaptive divergence in coral‐dwelling gall crabs: signature of host driven evolution. Chapter 10 in: Evolutionary diversification of coral‐dwelling gall crabs (Cryptochiridae). Ph.D. thesis, Leiden University, Leiden, The Netherlands.

[ece31808-bib-0036] van der Meij, S. E. T. , and B. W. Hoeksema . 2013 Distribution of gall crabs inhabiting mushroom corals on Semporna reefs, Malaysia. Mar. Biodivers. 43:53–59.

[ece31808-bib-0037] van der Meij, S. E. T. , and R. R. Visser . 2011 The *Acropora humilis* group (Scleractinia) of the Snellius expedition (1929–30). Raffles Bull. Zool. 59:9–17.

[ece31808-bib-0038] Montano, S. , D. Seveso , P. Galli , S. Puce , and B. W. Hoeksema . 2015 Mushroom corals as newly recorded hosts of the hydrozoan symbiont *Zanclea* sp. Mar. Biol. Res. 11:773–779.

[ece31808-bib-0039] Netchy, K. , P. Hallock , K. S. Lunz , and K. L. Daly . in press. Epibenthic mobile invertebrate diversity organized by coral habitat in Florida. Mar. Biodiv. doi: 10.1007/s12526‐015‐0388‐7.

[ece31808-bib-0040] Norton, D. A. , and M. A. Carpenter . 1998 Mistletoes as parasites; host specificity and speciation. Trends Ecol. Evol. 13:101–105. 21238220 10.1016/S0169-5347(97)01243-3

[ece31808-bib-0041] Paterson, A. M. , and J. Banks . 2001 Analytical approaches to measuring cospeciation of host and parasites: through a glass, darkly. Int. J. Parasitol. 31:1012–1022. 11406147 10.1016/s0020-7519(01)00199-0

[ece31808-bib-0042] Paulay, G. , R. Kropp , P. K. L. Ng , and L. G. Eldredge . 2003 The crustaceans and pycnogonids of the Mariana Islands. Micronesica 35–36:456–513.

[ece31808-bib-0043] Ronquist, F. , and J. P. Huelsenbeck . 2003 MrBayes 3: Bayesian phylogenetic inference under mixed models. Bioinformatics 19:1572–1574. 12912839 10.1093/bioinformatics/btg180

[ece31808-bib-0044] Scaps, P. , and V. Denis . 2008 Can organisms associated with live scleractinian corals be used as indicators of coral reef status? Atoll Res. Bull. 566:1–18.

[ece31808-bib-0045] Stella, J. S. , G. P. Jones , and M. S. Pratchett . 2010 Variation in the structure of epifaunal invertebrate assemblages among coral hosts. Coral Reefs 29:957–973.

[ece31808-bib-0046] Swofford, D. L. 2002 PAUP*: Phylogenetic Analysis Using Parsimony (* and other methods). Version 4.0b10. Sinauer Associates, Sunderland.

[ece31808-bib-0047] Takeda, M. , and Y. Tamura . 1979 Coral‐inhabiting crabs of the family Hapalocarcinidae from Japan. I. Three species obtained from mushroom coral, *Fungia* . Bull. Natl Mus. Nat. Sci Ser. A Zool. 5:183–194.

[ece31808-bib-0048] Thomas, J. 1993 Biological monitoring and tropical biodiversity in marine environments: a critique with recommendations, and comments on the use of amphipods as bioindicators. J. Nat. Hist. 27:795–806.

[ece31808-bib-0049] Westoby, M. 2006 Phylogenetic ecology at world scale, a new fusion between ecology and evolution. Ecology 87:S163–S165. 16922311 10.1890/0012-9658(2006)87[163:peawsa]2.0.co;2

