# Supplementary figures and images for: Phylogenetic ecology of gall crabs (Cryptochiridae) as associates of mushroom corals (Fungiidae)
Source: Ecol Evol. 2015 Nov 24;5(24):5770–80. doi: 10.1002/ece3.1808 (PMC4717343; doi:10.1002/ece3.1808)

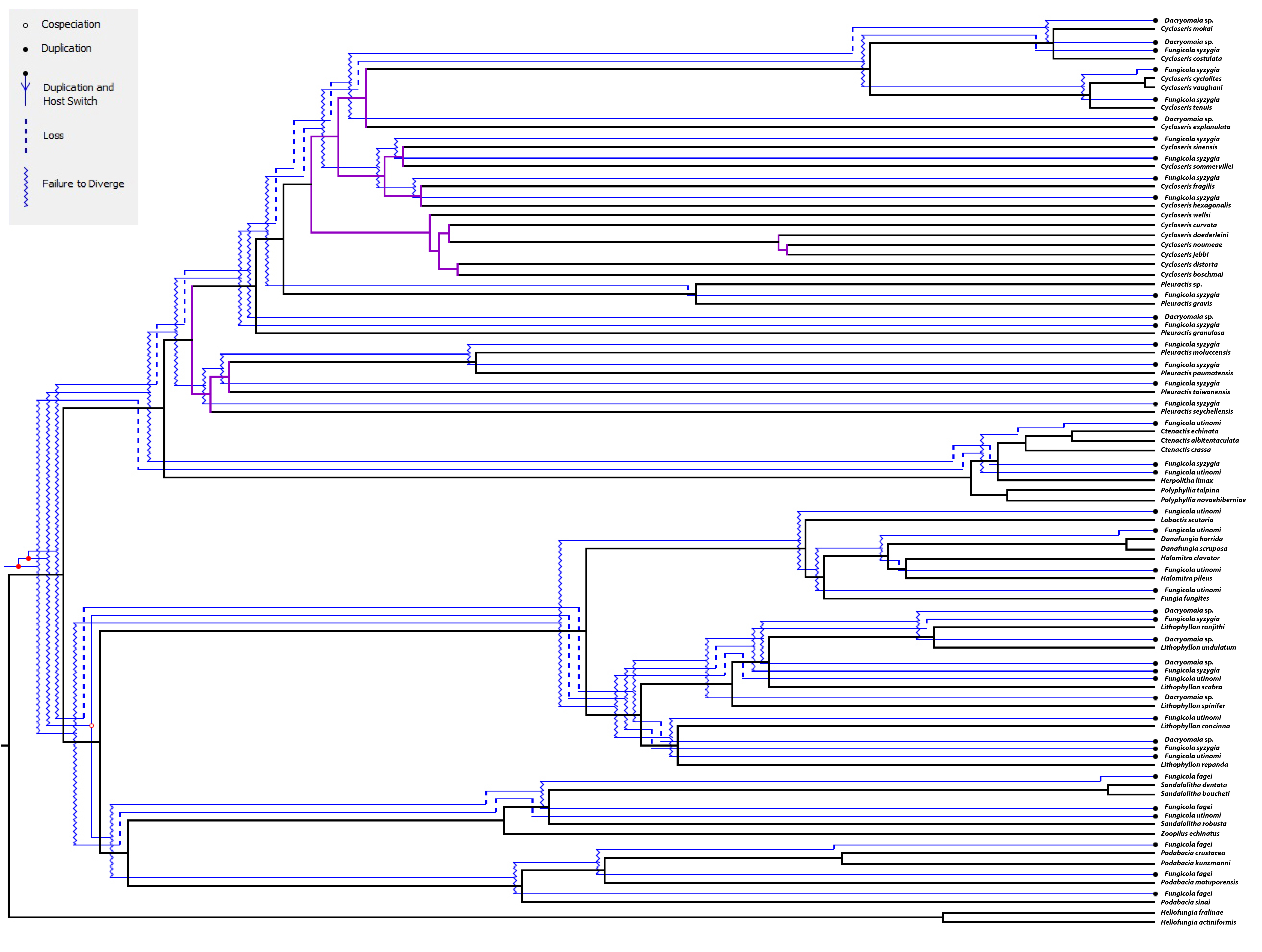

Supplement: Supplementary file 2 — Figure S1. Tree resulting from analysis in Jane 4.0 showing the different coevolutionary events between Fungiidae (black lines) and Cryptochiridae (blue lines), based on the complete dataset. [file ECE3-5-5770-s002.jpg]

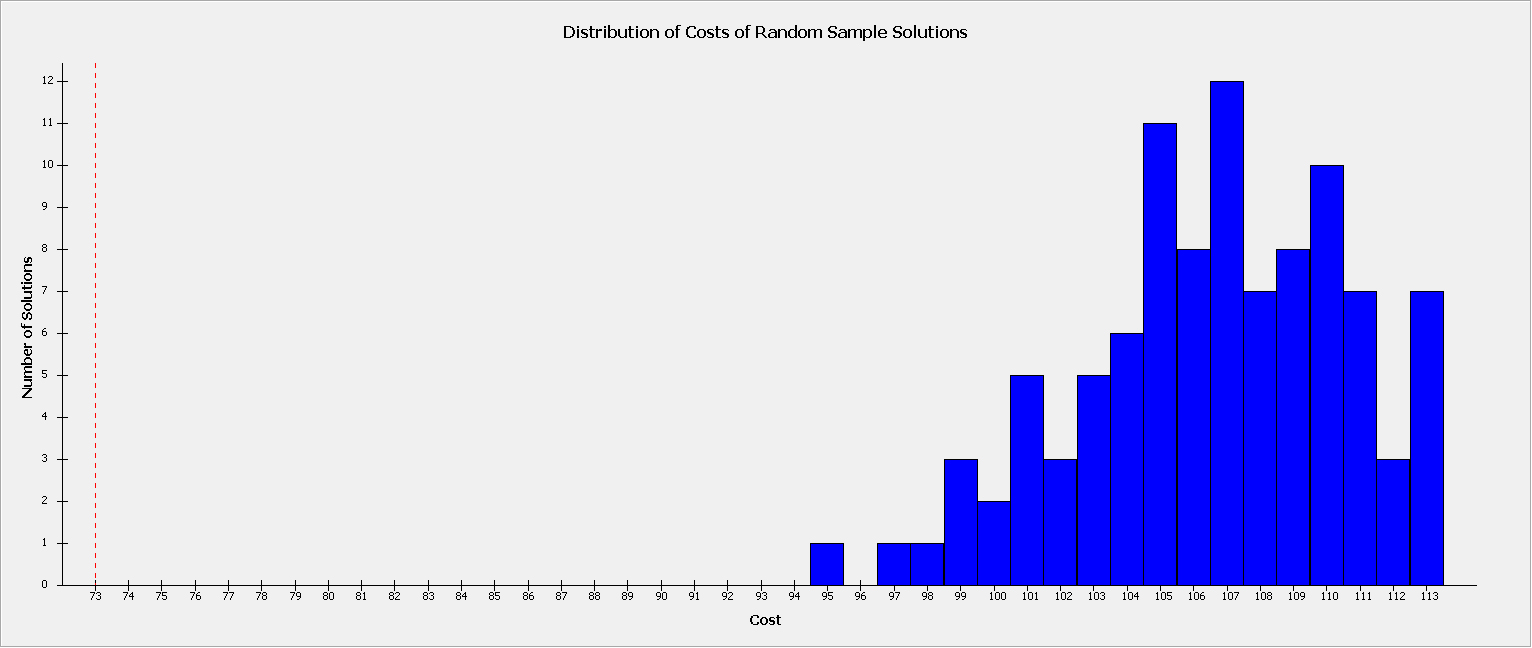

Supplement: Supplementary file 3 — Figure S2. Histogram resulting from a stats run in Jane 4.0 on the complete dataset, showing the distributions of costs of the random sample solutions. The costs of the optimal [=coevolution] solution is indicated by the red dotted line. [file ECE3-5-5770-s003.jpg]

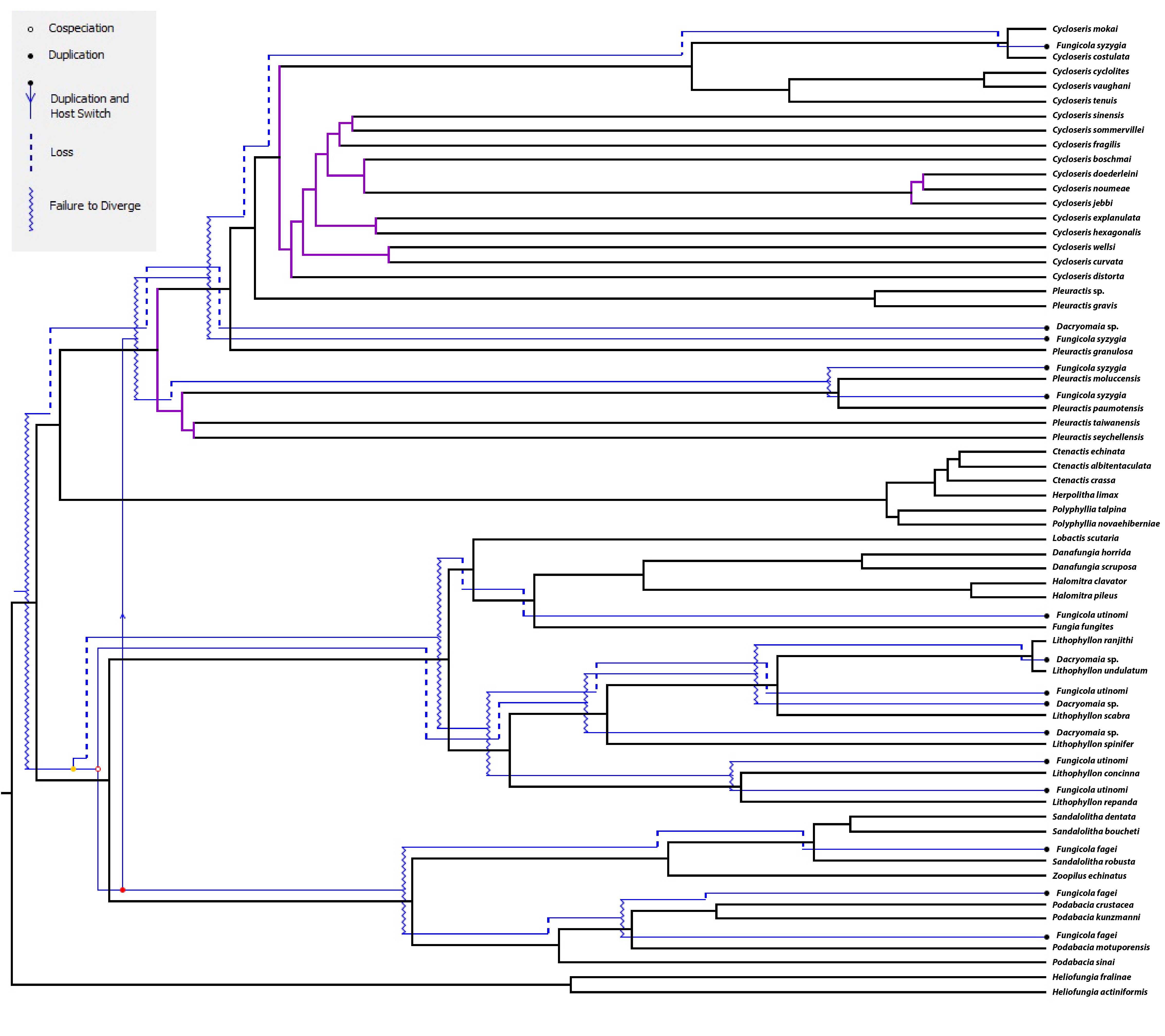

Supplement: Supplementary file 4 — Figure S3. Tree resulting from analysis in Jane 4.0 showing the different coevolutionary events between Fungiidae (black lines) and Cryptochiridae (blue lines), based on the common occurrences dataset. [file ECE3-5-5770-s004.jpg]

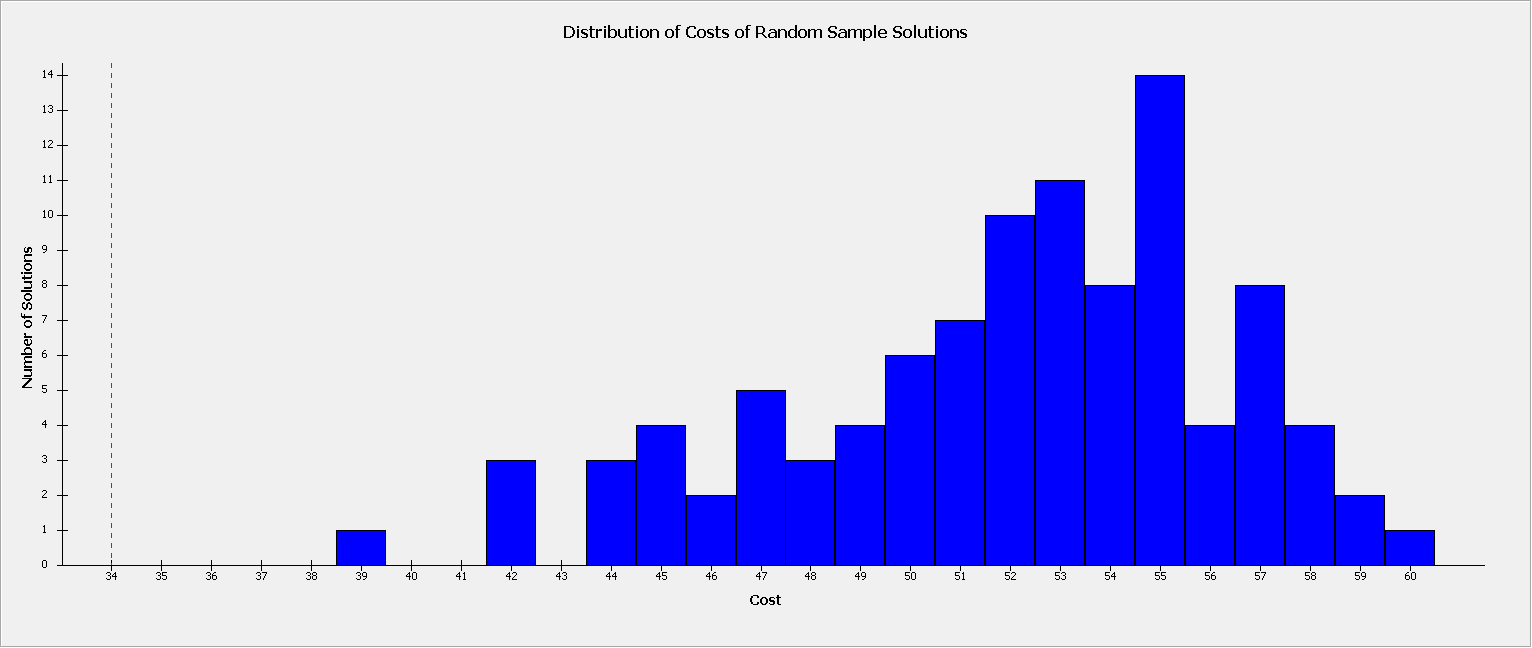

Supplement: Supplementary file 5 — Figure S4. Histogram resulting from a stats run in Jane 4.0 on the common occurrences dataset, showing the distributions of costs of the random sample solutions. The costs of the optimal [=coevolution] solution is indicated by the red dotted line. [file ECE3-5-5770-s005.jpg]
